# Woody plant secondary chemicals increase in response to abundant deer and arrival of invasive plants in suburban forests

**DOI:** 10.1002/ece3.8814

**Published:** 2022-04-13

**Authors:** Janet A. Morrison, Bernadette Roche, Maren Veatch‐Blohm

**Affiliations:** ^1^ 3280 Department of Biology The College of New Jersey Ewing New Jersey USA; ^2^ 28521 Department of Biology Loyola University Maryland Baltimore Maryland USA

**Keywords:** *Microstegium vimineum*, multiple stressors, plant defense, structural equation modeling, suburban forest, white‐tailed deer

## Abstract

Plants in suburban forests of eastern North America face the dual stressors of high white‐tailed deer density and invasion by nonindigenous plants. Chronic deer herbivory combined with strong competition from invasive plants could alter a plant's stress‐ and defense‐related secondary chemistry, especially for long‐lived juvenile trees in the understory, but this has not been studied. We measured foliar total antioxidants, phenolics, and flavonoids in juveniles of two native trees, *Fraxinus pennsylvanica* (green ash) and *Fagus grandifolia* (American beech), growing in six forests in the suburban landscape of central New Jersey, USA. The trees grew in experimental plots subjected for 2.5 years to factorial treatments of deer access/exclosure × addition/no addition of the nonindigenous invasive grass *Microstegium vimineum* (Japanese stiltgrass). As other hypothesized drivers of plant secondary chemistry, we also measured nonstiltgrass herb layer cover, light levels, and water availability. Univariate mixed model analysis of the deer and stiltgrass effects and multivariate structural equation modeling (SEM) of all variables showed that both greater stiltgrass cover and greater deer pressure induced antioxidants, phenolics, and flavonoids, with some variation between species. Deer were generally the stronger factor, and stiltgrass effects were most apparent at high stiltgrass density. SEM also revealed that soil dryness directly increased the chemicals; deer had additional positive, but indirect, effects via influence on the soil; in beech photosynthetically active radiation (PAR) positively affected flavonoids; and herb layer cover had no effect. Juvenile trees’ chemical defense/stress responses to deer and invasive plants can be protective, but also could have a physiological cost, with negative consequences for recruitment to the canopy. Ecological implications for species and their communities will depend on costs and benefits of stress/defense chemistry in the specific environmental context, particularly with respect to invasive plant competitiveness, extent of invasion, local deer density, and deer browse preferences.

## Graphical abstract

We tested the effects of overabundant white‐tailed deer and the nonindigenous, invasive grass *Microstegium vimineum* on foliar secondary chemistry of juveniles of the trees *Fraxinus pennsylvanica* and *Fagus grandifolia*, in six suburban forests of central New Jersey, USA. We analyzed leaves sampled from individuals growing in plots in a factorial experiment of deer exclosure presence/absence × *M. vimineum* addition/no addition. Using univariate mixed models and structural equation modeling, we found that in some cases these two common stressors in suburban forests increased juvenile trees’ secondary chemicals involved in defense and stress responses, deer generally had stronger and/or more consistent effects than *M. vimineum*, and additional influences on the trees’ secondary chemistry were revealed by the SEM, that is, light and soil moisture.

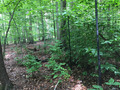

## 1 INTRODUCTION

Woodland plants within a suburban landscape live in circumstances that differ in many ways from rural environments with fewer anthropogenic influences (Morse et al., 2014), including the presence of many nonindigenous, invasive plant species (Aronson et al., 2015; Dolan et al., 2011) and very high white‐tailed deer (*Odocoileus virginianus* Zimmerman) densities (Urbanek & Nielsen, 2013). Fragmentation of suburban natural areas creates a high edge to interior ratio, creating many entry points for nonindigenous species (Cadenasso & Pickett, 2001; Hunter & Mattice, 2002) and rapid spread via trails and roads (Pickering et al., 2010; Schramm & Ehrenfeld, 2012). In suburban forests, the combination of forest patches with open areas is excellent deer habitat (Alverson et al., 1988; Potapov et al., 2014), while hunting is very limited (Williams et al., 2013) and most natural predators of deer are uncommon. These features of suburban forests cause plants to face the dual stressors of competition from spreading nonindigenous species and deer herbivory, but no studies have investigated plants’ chemical responses to these combined stressors. Here, we report on the foliar antioxidant, phenolic, and flavonoid responses in juveniles of two native tree species in forests of suburban New Jersey, USA.

The ability of plants to respond to biotic and abiotic stressors depends on regulatory networks that help balance resource allocation to growth or defense (Wu & Baldwin, 2009). Reactive oxygen species (ROS) increase during stress (Baxter et al., 2014; Del Río, 2015), causing oxidative destruction of cells, but this can be countered by antioxidants, which play a scavenging role and minimize plant cell damage (Das & Roychoudhury, 2014). Overall antioxidant production, or more specific categories of antioxidants such as phenolics or flavonoids (a type of phenolic), can act as proxies for the degree of stress experienced by plants (Ashraf et al., 2018; Gill & Tuteja, 2010). Phenolics and flavonoids have dual roles as antioxidants and inducible defenses; they defend plant tissues against future herbivory, scavenge ROS involved in signaling bursts as a result of wounding (Baxter et al., 2014; Wu & Baldwin, 2009), and play a role in a generalized stress response (Chalker‐Scott & Fuchigami, 1989). Thus, we may expect antioxidants in general, and phenolics and flavonoids in particular, to increase in suburban woody plants subjected to the dual stressors of invasive plants and chronic deer pressure.

Nonindigenous, invasive plants can broadly influence plant communities (Vilà et al., 2011) through direct effects, for example, strong competition for resources (Gioria & Osborne, 2014) and allelopathy from plant chemicals (Callaway & Ridenour, 2004; Kalisz et al., 2021), and indirectly via modifications of biotic factors such as microbial communities and natural enemies, or of abiotic factors such as light and moisture availability (Levine et al., 2003; Skurski et al., 2014). How such impacts from invasive plants, in particular, may influence secondary chemistry of resident plants has not been studied. However, plant competition in general causes various stress responses, with increased antioxidants (Afifi & Swanton, 2012; Miranda‐Apodaca et al., 2020), phenolics (Darmanti et al., 2018; Fernandez et al., 2016), and flavonoids (Hazrati et al., 2021; Rockenbach et al., 2020), or alteration of the overall metabolomic profile (Gidman et al., 2003). Exposure to competitors’ allelopathic chemicals also can alter a plant's secondary chemistry (Fernandez et al., 2016; Gniazdowska & Bogatek, 2005). Therefore, competition from nonindigenous, invasive plants, especially those with allelopathic effects, could elicit strong chemical responses in the native community. Negative trade‐offs between defense and competitive ability also are possible (Ballhorn et al., 2014; Viola et al., 2010), so a resident plant faced with a new plant invader may be particularly vulnerable due to both strong competition and the cost of chemical response to that competition.

Browsing by ungulates also can broadly influence plant communities. White‐tailed deer are selective generalists (Swihart & Bryant, 2001), but exhibit an array of preferences for woody species, which can influence recruitment (Côté et al., 2004; Russell et al., 2001), shift canopy composition (Walters et al., 2020), and extirpate rare species (Côté et al., 2004; Griggs et al., 2006). Browsing on woody plants can lead to the induction of defense chemicals; phenolics (Nosko & Embury, 2018; Ohse et al., 2017) and flavonoids (Ohse et al., 2017) have been shown to increase after damage. Defense chemicals can reduce palatability to deer (Bee et al., 2011; Champagne et al., 2020), but they also can be correlated with slower growth rates (Augustine & McNaughton, 1998) due to trade‐offs between growth and defense (Herms & Mattson, 1992), which can leave plants vulnerable as they remain within the reach of deer (Vila et al., 2002).

Recent work compares the ecological effects of nonindigenous plant invasion and deer pressure on native communities (Blossey & Gorchov, 2017; Gorchov et al., 2021), but has not compared the chemical responses of native plants to both stressors. Given the protective role of plant secondary chemistry, but also its possible physiological cost (Ballhorn et al., 2014), such a comparison will aid our understanding of the relative importance of invasive plants and abundant deer in suburban plant communities. We hypothesized that both would prompt increased production of antioxidants, phenolics, and flavonoids in woody plants in our experiment, with the greatest responses under both stressors together, but we posed no a priori hypothesis about their relative importance.

**FIGURE 1:**
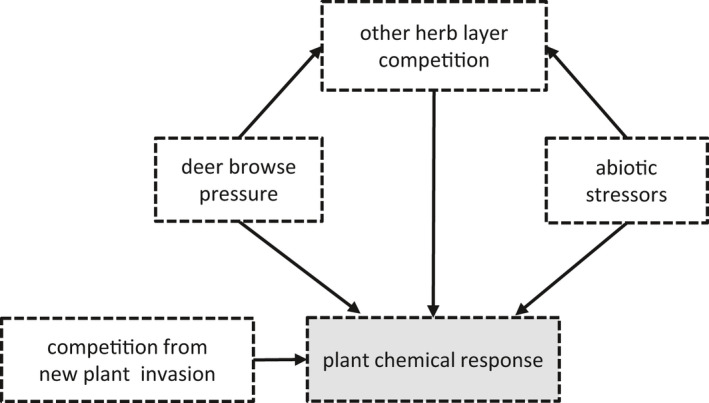
Structural equation meta‐model (SEMM), a system‐wide hypothesis of theoretical, interconnected drivers of woody plant chemistry in suburban forests

The analysis of ecological experiments benefits from combining univariate methods with multivariate structural equation modeling (SEM) (Grace, 2006, pp. 233–258) that focuses on system‐wide responses (Grace et al., 2009; Lamb & Cahill, 2008). We therefore also proposed a system‐wide hypothesis (Figure 1), represented as a structural equation meta‐model (SEMM). This hypothesis predicted that plant chemical responses would be increased by deer pressure and a new invader, as presented above, but additionally would increase due to direct effects from competition with the rest of the herb layer and from abiotic stressors known to influence secondary chemistry, specifically excessive light (Agati et al., 2012; Brunetti et al., 2015) and low soil moisture (Fini et al., 2012; Reddy et al., 2004). We also hypothesized that deer pressure and abiotic stressors would indirectly decrease the chemical responses via negative direct effects on the herb layer. For example, if herb layer plants declined due to an abiotic stress like drought, then there would be less stress from competition and a decreased chemical stress response in the target plants experiencing that competition. We limited the new invader's hypothesized effect to just that on plant chemistry because we had not observed any strong relationships between the manipulated invasive species in the experiment, *Microstegium vimineum* (Trin.) A. Camus (Japanese stiltgrass), and the other variables in the model.

## 2 MATERIALS AND METHODS

### 2.1 Study sites and species

Experimental plots (16 m^2^) were located in six forest stands within a suburban region of central New Jersey, USA, in Hopewell and Princeton Townships, Mercer County. The 131‐ to 174‐year‐old stands consist of closed canopies of mixed deciduous trees. The dominant canopy species in the forests are maples (*Acer rubrum*, *A*. *saccharum*), oaks (*Quercus rubrum*, *Q*. *velutina*, *Q*. *alba*, *Q*. *prinus*), hickories (*Carya* spp.), tulip poplar (*Liriodendron tulipifera*), American beech (*Fagus grandifolia*), green ash (*Fraxinus pennsylvanica*), sour gum (*Nyssa sylvatica*), and sweet gum (*Liquidambar styraciflua*) (Morrison et al., 2021). Their soils are silt loam or loam with 0–12% slopes (Natural Resources Conservation Service Web Soil Survey). Distance sampling conducted by professionals from the New Jersey Division of Fish & Wildlife and experienced hunters estimated deer density in the area at 32 deer/km^2^ (Hopewell Valley Deer Management Task Force, 2014), exceeding or similar to densities in studies that have shown significant influences on the vegetation of other eastern deciduous forests similar in species composition to the forests we studied (Aronson & Handel, 2011; Augustine & deCalesta, 2003; Horsley et al., 2003; McGarvey et al., 2013). The forests represent a sample of the fragmented forest parcels in the region, and display a range of ambient deer pressure. We did not have specific deer density measures for these small forest parcels since deer move through them, but we have characterized the forest‐specific deer pressure in Table 1, by the presence of hunting, shrub cover, herb layer native species richness, presence of oak juveniles, and a deer browse index (see Table 1 footnote for details).

**TABLE 1:** Deer pressure‐related forest characteristics

Forest	Years of hunting^a^	Percent native shrub cover^b^	Herb layer native species richness^b^	No. plots with red/black oak juveniles in spring, fall^c^	Percent browse index^d^
Baldpate (BAL)	12	56 (4)	22 (0.9)	18, 17 (IV = 33.1, #3)	0.39% (out of 520)
Nayfield (NAY)	5	27 (4)	13 (0.5)	18, 22 (IV = 84.7, #2)	2.3% (out of 442)
Herrontown (HER)	17	15 (3)	21 (0.8)	9, 10 (IV = 32.4, #5)	2.1% (out of 280)
Eames (EAM)	5	6.2 (3)	7.9 (0.3)	6, 0 (IV = 16.0, #5)	6.3% (out of 160)
Curlis (CUR)	0	2.5 (0.9)	6.8 (0.4)	4, 5 (IV = 94.0, #2)	10% (out of 228)
Rosedale (ROS)	0	0.55 (0.4)	8.7 (0.4)	2, 1 (IV = 29.8, #4)	6.8% (out of 177)

Note

All variables except hunting were measured in 32–40 16 m^2^ plots per forest. Values for shrub cover and species richness are the mean and SE. All data were from 2012, except percent browse was for species that were browsed in 2015 (with total sampled plants in parentheses). The canopy importance values for red + black oak are shown in parentheses, followed by the ranking of their importance value (IV) in that forest.

^a^ Hunting history was provided by Hopewell Valley Friends of Open Space and the Mercer County Parks Department, the owners and managers of these natural areas. These preserves are all near residential communities and hunting had been banned, but was eventually reinstated for deer management purposes. At the time of this study, Curlis and Rosedale had not yet been included in a deer management program.

^b^ Native shrub cover and herb layer native species richness decrease with deer overabundance (Rawinski, 2008). Shrub cover was measured with a “forest secchi” method (from Michael Van Clef, Hopewell Valley Friends of Open Space). It quantifies the percent vertical foliage cover of native woody plants in the deer browse zone, 0.4 m–1.4 m from the ground (Pierson & deCalesta, 2015), by a researcher observing from across the plot a 1 m^2^ board that was divided into a 4 x 4 grid, and counting the percentage of grid squares intercepted by native woody plants. This was done in two perpendicular directions and the values were averaged. Native species richness was from a spring herb layer census, using the census method described in the paper; the values shown are for the number of species in the 16 m^2^ plots.

^c^ *Quercus rubra* and/or *Q*. *velutina* (red and black oak) were the only preferred deer food species (Wakeland & Swihart, 2009) that also are common seed‐source canopy trees in each of this study's forests. *Quercus* presence was from spring and fall censuses. Canopy tree importance values for *Q*. *rubra* plus *Q*. *velutina* were obtained with standard procedures (Brewer & McCann, 1982).

^d^ The presence of tell‐tale shredded twig tips indicated deer browse (Pierson & deCalesta, 2015). The browse index for each forest consisted of the proportion of browsed individuals in unfenced plots of five native species that were browsed by deer and sufficiently common in the forests’ understories to use for comparison between forests: *Carya* spp., *Fagus grandifolia*, *Fraxinus pennsylvanica*, *Acer rubrum*, and *Rubus allegheniensis*.

The two native, woody species that were the subject of this foliar chemistry study were *Fagus grandifolia* Ehrh. (American beech) and *Fraxinus pennsylvanica* Marsh. (green ash). Both were common enough in the herb layers of the forests for our investigation, with the exceptions that Curlis Lake Woods had insufficient ash and Nayfield Preserve had insufficient beech to be included. A 2015 deer browse survey we did in the forests showed that both species were browsed by deer, with 16.8% of beeches (total *N* = 143) and 1.4% of ashes (total *N* = 559) exhibiting the tell‐tale shredded twig tips indicative of deer browse (Pierson & deCalesta, 2015). Study of both beech and ash allowed for consideration of the relative impact of deer preference on foliar chemistry.

It is worth noting that deer preferences and browse rates can vary widely among regions. Therefore, the browse rates measured in our central New Jersey forests should be seen as specific to our study and not applicable to other forests, which likely have lower or higher browse rates on beech and ash. For example, one review of beech ecology reflected the view that deer rarely feed on beech (Nyland et al., 2006). Other studies have shown 40% deer browse rates on beech and ash (Sedio et al., 2020), 18% on beech (Krueger et al., 2009), a range from 0% to 11% on beech depending on the site characteristics (Crimmins et al., 2010), and widely variable per‐plant browse intensity for both species (Liang & Seagle, 2002).

### 2.2 Experimental design

In each forest, 32–40 16 m^2^ plots were arranged on a grid with 4 m between plots. Each plot was randomly assigned a fencing or no‐fencing treatment and a stiltgrass seed addition or no‐addition treatment. The fences were installed in spring 2013. They were 2.3 m tall, consisting of plastic material with 4 × 4.5 cm mesh, made for deer exclosures (Deerbusters.com). The fencing was staked to the ground but had three cut‐outs at ground level on each side. This allowed entry by rabbits and voles and ensured that the only excluded herbivore would be deer. This fencing has no effect on light or wind speed (Morrison & Brown, 2004). Any leaf litter that accumulated against the fences in the border was removed twice per year, and vines that began to grow up the fences were clipped away as needed.

The stiltgrass seed addition treatment was applied in the fall of 2012. Each addition plot received 2.95 g of locally collected, pooled seeds (approximately 2420), mixed with 75 ml sand for easier distribution, after which the leaf litter and the soil surface were disturbed with a stout stick, allowing the seeds to settle down onto the soil surface (the no‐addition plots were disturbed in the same manner). We used this randomly assigned stiltgrass addition treatment to avoid any confounding site effects that could be associated with naturally occurring stiltgrass abundances. The seed additions were done after gaining permission from the forest preserve owners. Stiltgrass was not present in the specific study sites prior to the experiment, but was common elsewhere in the forests, as in nearly all forested areas of central New Jersey (personal observations). It is important to note that stiltgrass was removed where it appeared in the study sites outside of addition plots, and when ongoing research in the sites is concluded, it will be removed from addition plots until the seed bank is depleted. Subsequent recruitment and persistence of the introduced stiltgrass was highly variable among forests and plots, providing a range of densities that aligned with those found in naturally occurring stands in these forests: from nearly zero to nearly 100% cover.

We manipulated stiltgrass, specifically, because it is one of the most common and abundant invasive herb layer species in the region, and it has many documented negative effects on invaded plant communities (Adams & Engelhardt, 2009; Aronson & Handel, 2011; Flory & Clay, 2010; Oswalt et al., 2007). However, no research exists on its possible effects on indigenous plants’ foliar chemistry. There were other, naturally occurring, nonindigenous, invasive plant species present in all of the forests and many of the plots, but they varied among the forests and most were shrubs with low percent cover. The only herbaceous invasive plant with substantial cover was Japanese honeysuckle (*Lonicera japonica)*, but the most cover it had in any plot was only 9%, and its average cover was 0.8% and the median cover was zero.

### 2.3 Leaf collection

All leaves from beech and ash used in the study were collected on 2 Sept 2015. The number of plots sampled from each forest varied, based on the presence of beech and ash. In order to avoid biasing the results by tree age/size, in each sampled plot leaves were collected from one juvenile plant in each of three distinct size classes, as possible based on availability. If multiple plants in a size class were present, they were numbered and a random number generator dictated the choice. For beech, the size classes were: 0–10 cm, 20–40 cm, 50–140 cm. For ash, they were: only one set of simple leaves present, compound leaves with stem height ≤20 cm, compound leaves with stem height >25 cm. The two most distal (youngest) leaves were removed from all ashes and from unbranched beeches; for branched beeches, the most distal leaf on the lowest branch and the terminal branch was used. Leaves were collected from beech in five forests (not Nayfield), from 18 to 35 plots per forest and 19 to 53 plants per forest, with 101 plants from fenced plots and 83 from unfenced plots. Ash leaves were also taken from five forests (not Curlis), including 18 to 39 plots per forest and 30 to 95 plants per forest, with 163 in fenced plots and 156 unfenced. The two leaves from one plant were put into one envelope and then dried at 50°C for 3 days, in preparation for chemical analysis.

### 2.4 Foliar chemical analysis

We measured three categories of nonenzymatic antioxidants, from most to least inclusive: total antioxidants, total phenolics, and total flavonoids. Leaf samples (30 mg ± 0.1 mg dry weight) were taken from multiple parts of the leaf for both leaves within a sampled plant. The leaf samples were mixed with clean sea sand in a 1.5 ml microcentrifuge tube and ground into a fine powder before extraction with 1.52 ml of methanol. The tube was vortexed for 10 s; then the samples were put in a shaker at 150 rpm at 25°C for 60 min. The samples were then centrifuged for 5 min at 1118 *g*, followed by removal of the supernatant. Assays for antioxidant capacity, phenolic concentration, and flavonoid concentration were conducted on the supernatant.

Antioxidant capacity was analyzed in a 48 well plate using the ferric reducing ability of plasma (FRAP) assay, according to Benzie and Strain (1996). In brief, 900 µl of FRAP reagent was added to 30 µl of sample and 90 µl of ultrapure water, incubated for 4 min, and absorbance read at 593 nm on UV‐Vis spectrometer. The standard curve was generated using Trolox from 0 to 1500 µmole per liter. Antioxidant capacity of the samples is expressed as Trolox Equivalents (TE) per gram dry weight.

Phenolic concentration was tested using the Folin–Ciocalteu method (Ozsoy et al., 2008). In brief, 20 µl of the sample was mixed with 60 µl of Na2CO3, 900 µl of ultrapure water and 20 µl of three‐fold diluted Folin–Ciocaltue reagent. The samples were then vortexed and left to sit at room temperature for 2 h. Absorbance was read at 760 nm. Gallic acid (0 to 0.4 mg per ml) was used to generate the standard curve. The phenolic concentration of the samples is expressed as gallic acid equivalents (GAE) per gram dry weight.

Flavonoid concentration was analyzed in a 48 well plate using the aluminum chloride precipitation (Shams Ardekani et al., 2011). A sample volume of 100 µl was added to 400 µl of ultrapure water, then 30 µl of NaNO2 was added and allowed to sit for 5 min, followed by addition of 30 µl of AlCl3. After 1 min, 400 µl of NaOH was added. The absorbance was immediately measured at 510 nm. (+)‐Catechin (0–1000 ppm) was used to generate the standard curve. Flavonoid concentration of the samples is expressed as Catechin Equivalents (CE) per gram dry weight.

### 2.5 Field data collection

The proportion cover of all herb layer plants was quantified in each plot before leaf drop in the fall of 2015. Each species’ cover was scored as <1%, 1%–10%, 11%–20%, 21%–30%, etc. (in 10% intervals up to 100%) in 0.25 m^2^ quadrat frames, which were dropped without looking into each 1 m^2^ section of the 16 m^2^ plot. The score was converted to the interval's midpoint, and the mean of the 16 values provided one cover value per plot for each species, including stiltgrass. The values for all other species were summed to calculate the cover for all nonstiltgrass plants in the plot.

Photosynthetically active radiation at ground level was measured in each plot with a 1‐m‐long ceptometer (AccuPAR model PAR‐80 by Decagon Devices, Pullman, WA, USA). Measurements for a plot were made under cloudless conditions between 10 am and 2 pm of one day, at the four corners and center of each plot, and in nearby fields for full‐sun measures. Percent of full‐sun PAR was calculated for each plot by dividing the average of the five in‐plot readings by the full‐sun values from the same time point, and multiplying by 100. The measurements were done from 16 July to 20 October, as weather and schedules allowed, before leaf drop except for canopy ash trees (they were uncommon near the plots measured in October).

Soil water potential was measured as mPa with a bench‐top WP4 soil water potential meter (also Decagon Devices) on two soil samples taken from the top 3 cm of each plot on 14 September 2014. To capture conditions when variation in soil moisture could be detected, we ensured each collection was made when there had been a light rain the previous day (6 mm) and no rain for the six previous days.

We calculated an ambient deer browse index (DBI) for each forest to use in the SEMs. It consisted of the proportion of deer‐browsed individuals in unfenced plots of five native plant taxa: *Carya* spp., *F*. *grandifolia*, *Fraxinus pennsylvanica*, *Acer rubrum*, and *Rubus allegheniensis*. These were included because they were sufficiently common in the forests’ understories to allow for one index applicable to all of the forests and because they were, in our sites, neither the most browsed species nor completely avoided by deer. Other studies have used one sentinel species for a browse index (Blossey et al., 2019; Frelich & Lorimer, 1985; Koh et al., 2010). However, in our suburban forests with varying deer pressure and some very depauperate herb layers, no one species was suitable as a consistent indicator among forests. An index with multiple species offers a robust measure when species’ frequencies are highly variable among sites, as in our forests. Deer browse is readily identifiable. Deer have no upper incisors so they bite up on the stem, causing distinctive shredded tips, whereas a rodent clips the stem and leaves a clean, angled tip (Pierson & deCalesta, 2015). Deer browse data were collected in 16 to 20 unfenced plots per forests; within each plot all woody and semi‐woody individuals in a 0.5 × 7.5 belt transect were examined for the presence of deer browse.

### 2.6 Statistical analysis—Mixed models

We analyzed separate mixed models for antioxidant capacity, phenolic concentration, and flavonoid concentration, using PROC MIXED in SAS v 9.4 (SAS Institute Inc, 2015). Where enough plants were available, we collected leaves from three plants per species per plot for chemical analysis (in the plots where the species was present), but there were plots with just one or two plants of ash or beech. Therefore, the analyzed response variable was the mean value for all sampled individuals in a plot, thereby providing one value per plot. To normalize model residuals, all response variables were log_10_ transformed, except for ash flavonoids, which were square‐root transformed. All models were randomized complete blocks, with “forest” the random blocking factor (five forests). Fixed effects were “fencing” (either “fence” or “no fence”) and *Microstegium vimineum* (stiltgrass) percent cover (“mivi”), with four categorical levels based on the ranges of cover resulting from the experimental seed treatment: 0%, 0.03%–1.3%, 1.6%–5.6%, 12.2%–65%. Using these categories allowed us to test the idea that stiltgrass cover may have a threshold effect on foliar chemistry. The models also included the “fencing × mivi” interaction term. The omnibus tests were considered significant with an alpha critical value of 0.05. Because we had hypothesized that greater competition with the invasive species would increase the foliar chemicals in beech and ash, we did planned comparisons among all stiltgrass cover levels, using the Tukey–Kramer method to adjust for multiple contrasts and unequal sample sizes (Sokal & Rohlf, 1981). If the omnibus *p* value was >.05 but ≤.10, we still reported it to indicate a potentially causal relationship (Waite & Campbell, 2006) and conducted the planned multiple comparisons, following Ruxton and Beauchamp (2008).

The use of alpha critical values has been widely debated in ecology and statistics (Ellison et al., 2014). We have chosen to adhere to this still‐common practice in ecology (Mudge et al., 2012; Stanton‐Geddes et al., 2014) and note that the approach is reasonable for experimental studies like ours, in which treatment groups are randomly assigned (Johnson, 1999; Murtaugh, 2014). We acknowledge that the choice of 0.05 as a critical value, although still widely accepted and in use, is arbitrary and is weighted toward reducing Type I error over reducing Type II error. The cost of falsely rejecting the null hypothesis in our study (Type II error) we deem to be low since there are no important management, economic, or societal implications (sensu Hanson, 2011; Mapstone, 1995) of mistakenly concluding that deer and invasive species do not influence plant foliar chemistry. Therefore, we do not have any particular argument for increasing the alpha critical value and thereby decreasing Type II error. Rather, we prefer to retain the rigor of keeping Type I error at 0.05 to claim statistical significance, while being transparent in also reporting results with *p* < .10, which may be suggestive of effects from deer and invasive species. Finally, we do not rely solely on a statistical significance cut‐off, so we also report the actual *P* values and show means and 95% confidence intervals in order to indicate treatment effect sizes (Johnson, 1999; Murtaugh, 2014).

### 2.7 Statistical analysis—Structural equation modeling

**FIGURE 2:**
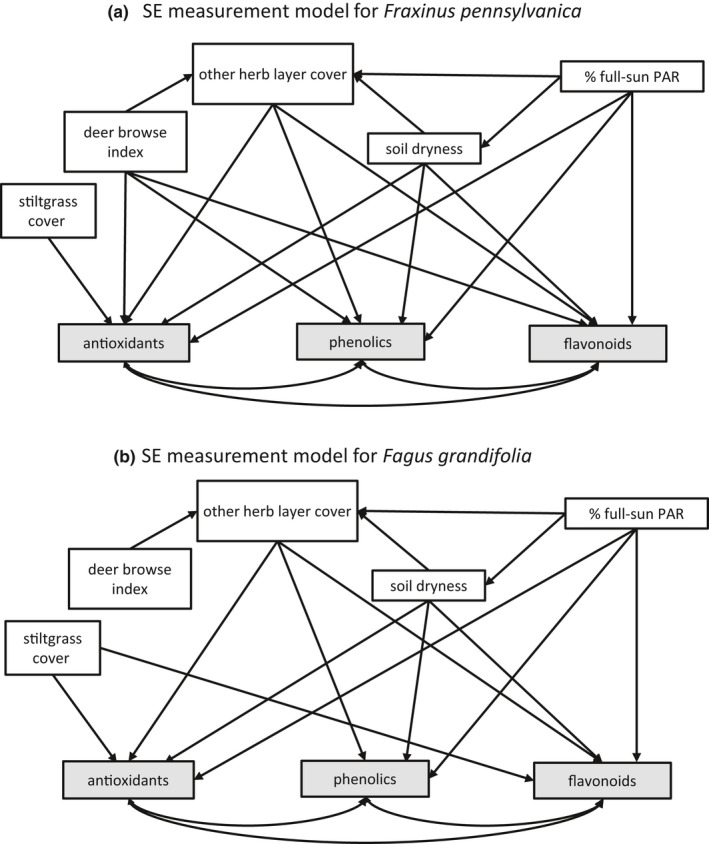
Initial structural equation measurement model, based on the SEMM of Figure 1 and guided by results from the univariate analyses

We conducted structural equation modeling with the “piecewiseSEM” package v. 2.0 (Lefcheck & Freckleton, 2016) in R v. 4.0.3 (R Core Team, 2020) using R Studio v. 1.2.5001. In this method, the psem() function was applied to the set of multiple linear regressions, built with lm(), that were specified in initial structural equation measurement models (Figure 2a and b) based on the proposed concepts and pathways in the conceptual SEMM (Figure 1) and informed by the results of the univariate mixed models. Specifically, the initial measurement models did not include paths from the deer browse pressure variable or stiltgrass cover to a chemical group if the fencing effect or stiltgrass cover effect was not significant in the mixed model. Using such prior knowledge of a system when developing an initial model is a key practice in structural equation modeling (Grace et al., 2010; Lefcheck & Freckleton, 2016). Antioxidants, phenolics, and flavonoids as described above measured the “plant chemical response” concept from the SEMM; the ambient DBI measured “deer browse pressure” in unfenced plots and was set to zero for fenced plots; a stiltgrass cover category, with four levels, measured “competition from new invasion”; the total nonstiltgrass proportion cover measured “other herb layer competition”; and soil dryness (−1 × soil water potential) and percent of full‐sun PAR measured ‘abiotic stressors’.

Note that stiltgrass cover and DBI were exogenous variables in the SEM, with no paths to them from other variables. This was because they were experimentally manipulated; by design, half of the plots had zero stiltgrass and the half that were fenced had zero deer browse pressure. Additionally, none of the mixed models indicated an interactive effect of deer exclosure fencing and stiltgrass cover on any of the foliar chemical groups, which supported not having any indirect paths from stiltgrass cover to the chemicals through the deer browse index, or vice versa.

All endogenous variables in the model were first transformed to better normalize the residuals from their regressions, which were checked by the Shapiro–Wilk statistic and with visualizations produced by the “fitdistrplus” package v. 1.1–1 (Delignette‐Muller & Dutang, 2015). Good transformations were indicated by the “bestNormalize” package v. 1.6.1 (Peterson & Cavanaugh, 2020), and included either log_10_ or square root transformations. In addition, prior to modeling, we removed several outliers and checked for any nonlinearities between variables by plotting the data, as recommended for SEM (Kline, 2015). In no case was it necessary to include nonlinear relationships in the SEM regressions. The Fisher's C statistic indicated model fit (Lefcheck & Freckleton, 2016). The modeling process was iterative. We began with the hypothesized measurement models in Figure 2a and b, then removed nonsignificant paths and added any significant and ecologically sensible paths that were indicated by psem() to be necessary for model fit.

## 3 RESULTS

### 3.1 Mixed models

#### 3.1.1 Ash

**FIGURE 3:**
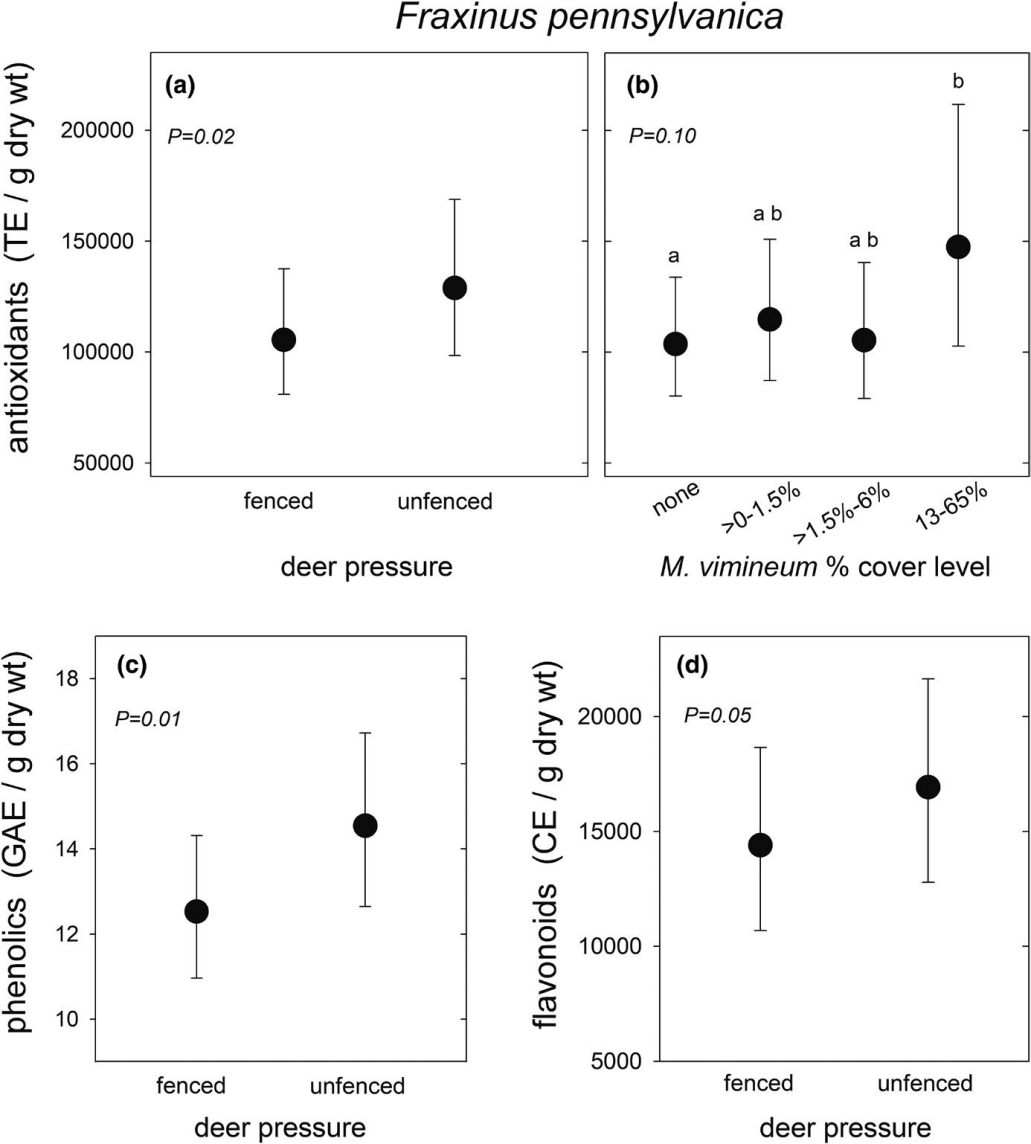
Total antioxidants (a), phenolics (b, c), and flavonoids (d) in leaves of *Fraxinus pennsylvanica* juveniles in central New Jersey, USA forests. Plants grew in fenced or unfenced plots (a, c, d) and with four levels of *Microstegium vimineum* cover (b). Graphs show least‐squares means ± 95% CL, backtransformed from log_10_ for antioxidants and phenolics and from square roots for flavonoids. *N* for each mean, from right to left: (a) 77, 75; (b) 84, 35, 24, 9; c and (d) 79, 77. Means labeled with different letters in B were different only at the *p* = .09 level, based on adjustment for six multiple comparisons with the Tukey–Kramer method

Foliar antioxidant concentration in ash plants was 22% greater on average in unfenced plots compared to fenced plots (Table 2, Figure 3a). Additionally, plots with the highest stiltgrass cover had, on average, 42% greater antioxidants than those with no stiltgrass, but this was not quite a significant effect at our critical value of 0.05 (Table 2, Figure 3b). There was no interaction between fencing and stiltgrass cover for ash antioxidants. Only the fencing treatment affected phenolics and flavonoids in ash, with 16% and 18% greater mean values, respectively, for plants in the unfenced plots (Table 2, Figure 3c, d).

**TABLE 2:** Mixed model results for the effects of fencing treatment, *Microstegium vimineum* (MIVI) cover, and interactions on foliar antioxidant capacity (a), phenolics concentration (b), and flavonoid concentration (c) in juveniles of the tree species *Fraxinus pennsylvanica* and *Fagus grandifolia* growing in forests of central New Jersey, USA

Source of variation	*F. pennsylvanica*			*F. grandifolia*		
	df (num, den)	*F*	*p*	df (num, den)	*F*	*p*
(a) Antioxidants						
Fencing	1, 140	5.7	.02	1, 105	0.04	.9
MIVI cover category	3, 140	2.1	.10	3, 105	2.8	.05
Fencing × MIVI cover	3, 140	0.40	.8	3, 105	0.3	.8
(b) Phenolics						
Fencing	1, 144	6.4	.01	1, 105	0.46	.5
MIVI cover category	3, 144	1.0	.4	3, 105	1.9	.14
Fencing × MIVI cover	3, 144	1.2	.3	3, 105	1.4	.3
(c) Flavonoids						
Fencing	1, 144	3.8	.05	1, 97	0.11	.7
MIVI cover category	3, 144	1.3	.3	3, 97	2.3	.08
Fencing × MIVI cover	3, 144	1.0	.4	3, 97	1.7	.2

#### 3.1.2 Beech

**FIGURE 4:**
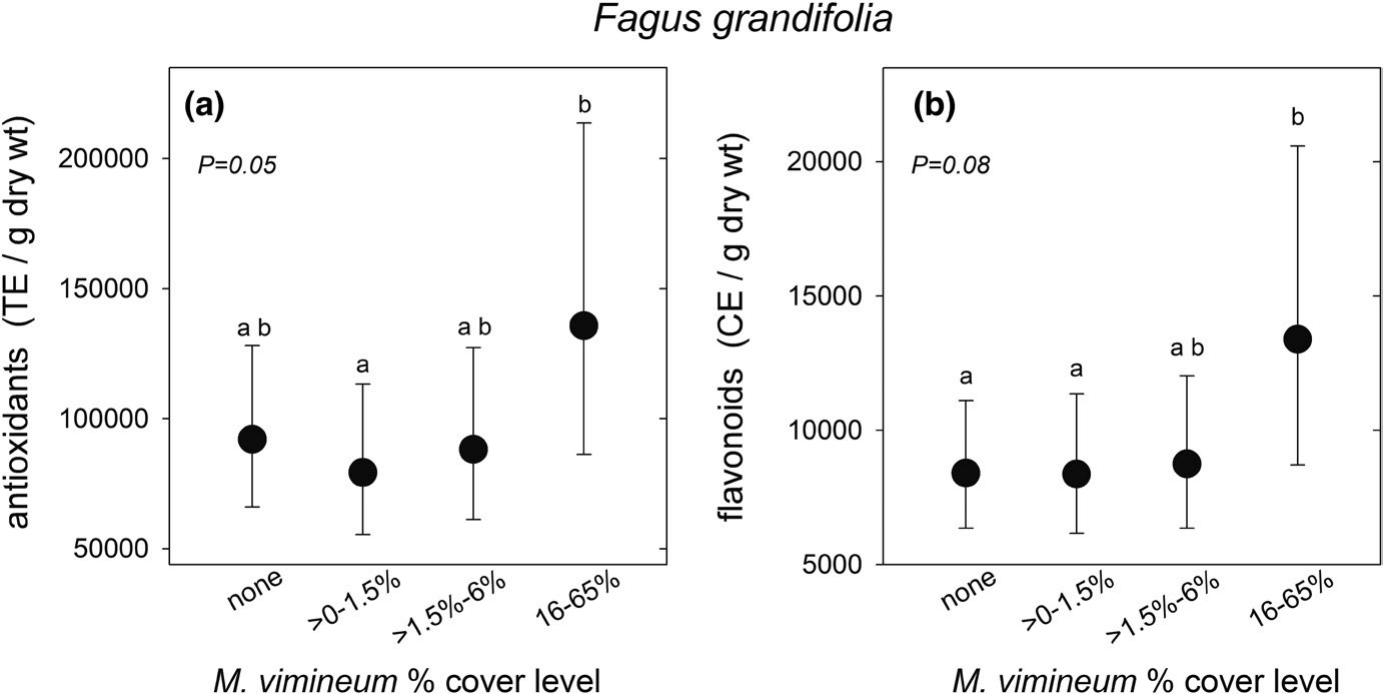
Total antioxidants (a) and flavonoids (b) in leaves of *Fagus grandifolia* juveniles in central New Jersey, USA forests growing with four levels of *Microstegium vimineum* cover. Graphs show least‐squares means ± 95% CL, backtransformed from log_10_. *N* for each mean, from right to left: (a) 61, 25, 22, 9; (b) 60, 13, 19, 7. Means labeled with different letters in A were different at *p* = .03 and in B they were different only at *p* = .06 (none vs. 16%–65%) and *p* = .07 (>0–1.5% vs. 16–65%) level, based on adjustment for six multiple comparisons with the Tukey–Kramer method

Antioxidants increased in beech plants that grew in plots with the highest stiltgrass cover level (Table 2); mean antioxidants were 71% greater in plots with the 12%–65% cover level compared to the >0–1.5% cover level (Figure 4a). Flavonoids were 59% and 60% greater in plots with the highest stiltgrass cover level, compared to no stiltgrass and >0%–1.5% stiltgrass, respectively, but these contrasts were slightly over the .05 critical value for significance (Figure 4b). Neither beech antioxidants nor flavonoids were affected by the fencing treatment nor its interaction with stiltgrass cover, and beech phenolics were not affected by deer, stiltgrass cover, or their interactions (Table 2).

**FIGURE 5:**
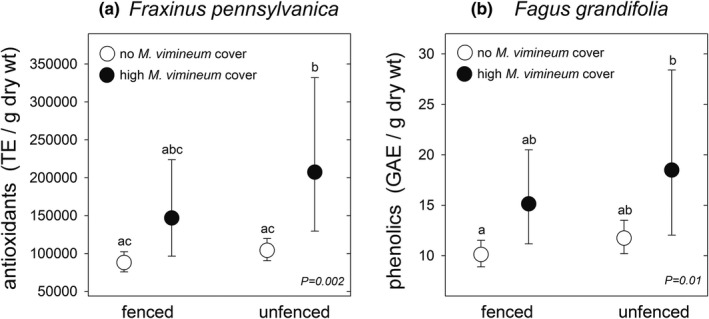
Total *Fraxinus pennsylvanica* antioxidants (a) and *Fagus grandifolia* phenolics (b) in leaves of juveniles in central New Jersey, USA forests growing in fenced or unfenced plots and with no *Microstegium vimineum* cover or high cover, defined as 12%–65%. Graphs show least‐squares means ± 95% CL, backtransformed from log_10_. *N* for each mean, from right to left: (a) 39, 4, 49, 4; (b) 33, 6, 28, 3. Based on adjustment for six multiple comparisons with the Tukey–Kramer method, means labeled in A with different letters were different at *p* = .005 (fenced/no cover vs. unfenced/high cover) and *p* = .03 (unfenced/no cover vs. unfenced/high cover). In b, they were different at *p* = .04 (fenced/no cover vs. unfenced/high cover)

One goal of the initial experimental design was to test the hypothesis that the dual stressors of competition from high stiltgrass cover and chronic deer browsing would cause the greatest increases in foliar secondary chemicals. This could have been indicated from significant fencing × stiltgrass cover level interactions, but none were detected in the full models. We had expected the stiltgrass seed addition treatments to result in uniformly high cover of stiltgrass, but this occurred only in a small number of plots scattered across the forests. Therefore, as another test of this hypothesis, for each species‐chemical combination we did a set of simple planned comparisons between four groups (pooled across the forests): fenced/zero stiltgrass cover, fenced/high stiltgrass cover, unfenced/zero stiltgrass cover, unfenced/high stiltgrass cover. High cover was defined as >12%–65%. For four of the six species‐chemical combinations there were no significant contrasts between any groups. However, ash antioxidant values were 136% and 99% greater in the unfenced/high cover group versus the fenced/zero cover group and the unfenced/zero cover group, respectively (Figure 5a), and beech phenolics were 83% greater in the unfenced/high cover group vs. the fenced/zero cover group (Figure 5b).

### 3.2 Structural equation models

#### 3.2.1 Ash

**FIGURE 6:**
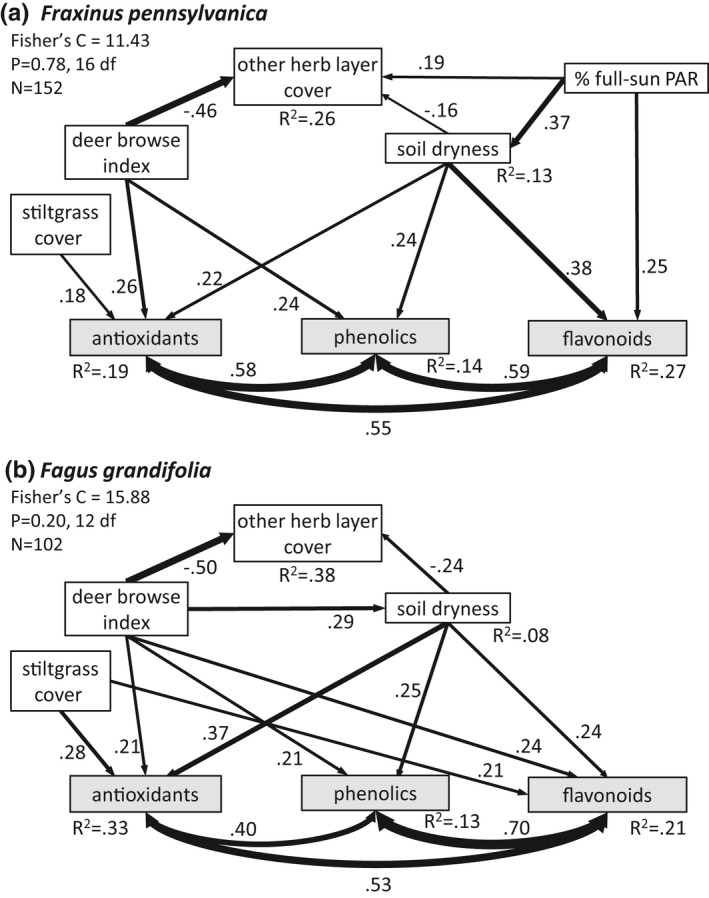
Fitted structural equation models of drivers of foliar plant secondary chemistry in juveniles of the trees *Fraxinus pennsylvanica* and *Fagus grandifolia*, growing in suburban forests of central New Jersey, USA. Path thickness is proportional to the values of the standardized path coefficient labels. All paths are significant at *p* < .05

We arrived at a final, fitted SE model (Figure 6a) that both reinforced many of the findings above for ash, and also provided additional insights. First, as in the univariate mixed models, the SEM revealed a strong, direct, positive effect of stiltgrass cover on antioxidants and no effect on phenolics or flavonoids. Second, as in the univariate models, deer browse pressure (measured as DBI) positively affected antioxidants and phenolics, but did not affect flavonoids, mirroring the somewhat weaker effect of fencing on flavonoids (*p* = .05 vs .01 and .02 for the other chemicals). DBI had a strong negative effect on herb layer cover, but there was no significant effect of the herb layer on any foliar chemicals. Third, the two abiotic variables in the ash SEM were very influential, with various strong direct and indirect effects on foliar chemistry, for example, greater concentrations of all three chemical types with increasing soil dryness and a direct positive effect on flavonoids from increased PAR.

#### 3.2.2 Beech

The final SEM for beech also provided many similar findings as the univariate models, along with some new and different results (Figure 6b). First, as in the univariate models, stiltgrass cover positively influenced antioxidants and flavonoids, but not phenolics. Second, DBI had direct, positive influences on all three chemical types, in addition to a net positive effect via the indirect pathway through soil dryness, which differed from the univariate analysis in which there were no significant effects of deer exclosure fencing. As in the ash SEM, deer had a very strong negative effect on the other herb layer vegetation, but that in turn had no paths to the beech chemical variables. Third, all chemical concentrations increased with greater soil dryness, but PAR did not have any effects and was dropped from the model.

Overall, the SEMs suggested that (1) deer and abiotic factors had greater influences on leaf chemistry than did the invasive species *M*. *vimineum* or competition from other plants; (2) although the three chemicals’ values were positively correlated, as expected, they did not respond identically to the variables and were more similar in the beech SEM; (3) a substantial amount of variation in the models remains to be explained by unmeasured factors.

## 4 DISCUSSION

### 4.1 Effects of stiltgrass cover

This study provided partial support for the hypothesis that a newly introduced, invasive, nonindigenous species can increase foliar antioxidants, phenolics, and flavonoids of plants in the invaded community. Support was shown in both types of analysis; there were positive paths from stiltgrass cover to ash and beech antioxidants and beech flavonoids in the SEM, and the univariate mixed model showed significantly greater beech antioxidants in plots with the highest stiltgrass cover level. In addition, there were several contrasts in the univariate models between the highest stiltgrass cover level and the zero or >0%–1.5% that were close to our significance level of .05, suggesting that high stiltgrass cover may have caused greater antioxidants in ash and flavonoids in beech. However, stiltgrass had no effects on phenolics in either species or flavonoids in ash, except indirectly in the SEM via correlations between the chemical groups. Ash and beech are mid‐ to late‐successional tree species, respectively (Burns & Honkala, 1990). They may remain as juveniles for years, and so must contend with long‐term competition from plants in the herb layer, which can be intense from a rapidly increasing invader (Gioria & Osborne, 2014), potentially limiting resources, depressing growth rates, and reducing a tree's chance of reaching the canopy. If the competition also induces increased production of secondary chemicals, as observed here in some cases, the plants may incur an added cost in even lower growth rates, given the possibility of growth‐defense tradeoffs. Indeed, such tradeoffs have been documented for induced defenses in woody species (Donaldson et al., 2006; Fernandez et al., 2016; Sampedro et al., 2011).

To our knowledge, no other studies have demonstrated induction of secondary chemistry by a nonindigenous, invasive species, including the well‐studied Japanese stiltgrass. Previous research indicates that it has allelopathic potential (Cipollini & Bohrer, 2016; Corbett & Morrison, 2012), and since secondary chemical responses to allelopathy have been shown in other systems, this is a possible mechanism worth further study. Stiltgrass's influence likely relies on it reaching a certain threshold of density during invasion; in our study its influence was generally due to its highest cover level.

The differences among the species‐chemical combinations for the effects of stiltgrass have several possible explanations. Plant secondary chemistry is influenced by a wide array of factors (e.g., resource availability, ontogeny), and variation in their production is common within populations (Hahn & Maron, 2016) and communities (Sedio et al., 2017). The datasets for each species came from a somewhat different set of forests and plots, so they may have experienced different resource conditions that mediated the competitive impact of stiltgrass. Competition intensity can alter chemical responses as shown, for example, in a study where specific flavonoids increased under low competition but decreased under high competition (Hazrati et al., 2021). The stronger influence of stiltgrass on flavonoids in beech versus ash could be due to beech's greater shade tolerance (Burns & Honkala, 1990). Its slower growth rate in the herb layer may allow it to invest more in secondary chemicals than the faster‐growing ash, as has been predicted (Coley et al., 1985) by the Resource Availability Hypothesis and shown (Endara & Coley, 2011), particularly for forest tree seedlings (Imaji & Seiwa, 2010). Total antioxidants in ash were directly affected by stiltgrass, but phenolics and flavonoids were not; it is likely the case that antioxidants other than phenolics were induced by stiltgrass competition. These differences could be resolved with a metabolomics approach in future research.

### 4.2 Effects of deer pressure

The hypothesis that deer pressure increases foliar concentrations of antioxidants, phenolics, and flavonoids was also partially supported for both species in this study. For ash, all three chemical groups were significantly greater in unfenced plots versus fenced plots, as shown by the univariate analyses. The SEM also indicated a positive effect of the deer browse index on ash antioxidants and phenolics, but not on flavonoids, except indirectly through the other chemical groups. For beech, a positive effect of deer on all three foliar chemical groups was apparent in the SEM, but the mixed models showed no significant effects from the fencing treatment. These induced chemical responses to deer pressure could have been recent or even months old, as long‐lasting effects on induced defenses have been shown previously for woody species (Lindroth et al., 2007; Nosko & Embury, 2018; Valkama et al., 2005), including in *Fagus* (Ohse et al., 2017) and *Fraxinus* (Friedman et al., 2020). The responses to deer can have two functions with ecological implications for browsed plants. On the one hand, they can become more protected against future browse, which should be very beneficial for growth and survival and could create an advantage in the plant community of suburban forests with high deer densities. On the other hand, if there is a substantial cost to induced defenses, a browsed woody plant could experience double jeopardy: loss of tissue coupled with lower growth potential that prevents it from escaping above the browse line. However, we cannot always assume a cost of induced defense (Steppuhn & Baldwin, 2008). Which scenario applies will depend on the relative costs and benefits, which rely on a complex suite of intersecting factors, for example, the level of herbivory pressure, competition, and tolerance traits.

Beech was browsed much more frequently than ash in the forests of this study, so we would expect it to have stronger secondary chemical responses to deer. This was indicated by the SEMs, but the mixed models suggested that ash was more affected. We have no specific explanation for this difference, except to note that SEM is a multivariate approach that is more representative of real ecological communities. Even so, given the low browse rate on ash in these forests, it seems to have mounted a strikingly strong chemical response to deer pressure.

### 4.3 Effects of plant invasion + deer pressure

The hypothesis that deer pressure and the invasive species together would cause the greatest foliar chemical responses was partially supported in this study. Although the availability of data for plots with high stiltgrass was limited, we still detected significantly greater ash antioxidants and beech phenolics in the plots with the dual stressors of deer access (unfenced) and high stiltgrass cover compared to the plots with neither stressor (fenced, zero stiltgrass), whereas high stiltgrass cover or unfenced treatment alone did not cause a significant increase in beech phenolics. However, there were no differences among the treatment groups for any of the other species‐chemical combinations, and no significant fencing × stiltgrass cover level interaction terms in the full mixed models. Still, these results illustrate that some woody plants experience an enhanced secondary chemical response when faced with multiple stressors. The roles of multiple stressors in biological systems are increasingly recognized across disciplines (Estravis‐Barcala et al., 2020; Orr et al., 2020), and has specifically been documented for deer pressure combined with earthworm invasions, non‐native plant invasion, and herbivory by rodents (Blossey et al., 2017; Dobson et al., 2020; Fisichelli & Miller, 2018).

### 4.4 Relative strengths of plant invasion and deer pressure effects

We sought to determine which factor—plant invasion or deer pressure—had greater influence on plant secondary chemistry. The SEMs suggest that, in this study, deer pressure was the more important factor. It had direct positive influences on nearly all of the chemical groups. Additionally, greater deer pressure in the SEM increased soil dryness in the beech SEM, which in turn increased antioxidants, phenolics, and flavonoids. In contrast, no strong indirect paths from stiltgrass to the chemicals were apparent, and while the strengths of the significant direct paths from stiltgrass cover to the chemical variables (0.18, 0.28, 0.21) were similar to those from the deer browse index variable (0.26, 0.24, 0.21, 0.21, 0.24), there were fewer of these direct paths. The univariate analyses were mixed on this point, showing stronger effects of deer on ash foliar chemicals, but stronger effects of stiltgrass cover in beech.

A recent review of published deer‐invasive plants experiments (Gorchov et al., 2021) concluded that deer are generally a more influential factor in deciduous forest communities of eastern North America than are invasive plants. Our research provides a new dimension to this comparison: for at least some woody species, deer pressure likely placed greater demands on plant secondary chemistry than competition from an invading plant. Even so, it is perhaps more important to recognize that both stressors induced responses in both species, and with a more widespread invasion, stress from stiltgrass competition likely would increase. In our study, effects from free‐ranging deer were likely much more spatially homogenous and widespread than that of the patchy *M*. *vimineum*, which invaded some plots much more readily than others.

### 4.5 Dual analysis: Univariate models and structural equation modeling

The two different analytical approaches used in this study complemented each other (Massad et al., 2017; Sudnick et al., 2021) and provided a more holistic picture of what is driving the induction of foliar chemistry in juveniles of two woody species. The experiment was designed to test for the main effects of and interactions between deer exclosure fencing and stiltgrass cover, as in any standard factorial design. The univariate analyses revealed these effects, but they also helped to guide the development of the initial SE measurement model. In turn, the fitted SEMs provided additional insight into the univariate results. Specifically, they confirmed the positive influences of stiltgrass cover only on antioxidants for ash and, for beech, on just antioxidants and flavonoids. However, it turned out that significant paths did exist between the deer browse index and all of the beech chemical groups. The fencing effect in the mixed models only compared fenced and unfenced plots, without taking into account any important variation in ambient deer pressure among the forests, which could affect the unfenced plots. The SEMs’ deer browse index, however, was modeled in a regression context, with deer browse pressure estimates for the unfenced plots that were distinct for each forest, and this variation in ambient deer browse pressure was important for most of the foliar chemicals. The larger context of the SEM also allowed for consideration of the relative importance of the experimental treatments when modeled alongside other drivers in the system. For example, stiltgrass cover significantly increased antioxidants in beech in the mixed model, but in the SEM its positive path was weaker than the effect of droughty soil.

### 4.6 Other variables as drivers in the SEM: herb layer cover, soil dryness and PAR

Not surprisingly, in both ash and beech SE models, there was a strong negative effect of deer on the nonstiltgrass herb layer cover. We had hypothesized in the structural equation meta‐model that, in turn, this reduced cover would cause less competitive stress on beech and ash juveniles, thereby decreasing their antioxidants, phenolics, and flavonoids. This would therefore have revealed a positive, indirect effect of deer on the foliar chemistry. However, the SEM did not show any effects on beech and ash foliar chemistry due to the nonstiltgrass cover. This contrasts to the positive effect that stiltgrass cover had, suggesting that greater stress was caused by the invasive species.

Soil dryness had direct, positive effects on each of the three chemicals in the SEMs, for both species. Levels of antioxidants are generally increased under drought stress (Reddy et al., 2004). For example, studies of *Quercus ilex*, which shares beech's family (Fagaceae), found increased phenolic production under drought conditions (Rivas‐Ubach et al., 2014), and flavonoids have been proposed as a secondary antioxidant system activated in severe stress (Agati et al., 2012; Fini et al., 2012). Beech and ash exhibited somewhat different strengths of their chemical responses to drought stress, which is not surprising. Within *Quercus*, for example, three species had different foliar concentrations of antioxidants in response to drought (Bilska et al., 2019), and even within a species local adaptation can result in different strategies for drought stress tolerance (Du et al., 2016).

Photosynthetically active radiation (PAR) significantly, positively affected flavonoids in ash, but not in beech. High PAR can lead to excess excitation energy, resulting in reactive oxygen species (ROS) production that may cause damage to photosystems I and II. ROS produce signaling cascades that adjust metabolism using a variety of stress‐protective mechanisms, including nonenzymatic antioxidants (Vuleta et al., 2015). For example, excess light is known to upregulate the production of flavonoids, which act as ROS scavengers (Agati et al., 2012) and are involved in photoprotection (Ryan et al., 2001). In the ash SEM, PAR also had indirect, positive effects on all three chemical groups through its positive effect on soil dryness, which in turn had positive effects on the chemical levels.

The SEMs were designed to include major factors that we hypothesized to be important drivers of plant secondary chemistry in the forests. They revealed a number of significant paths, but the explained variation (*R* ^2^ values) for the three chemical groups ranged only from 0.13 to 0.33. Intraspecific variation in specific types of secondary metabolites and the overall metabolome is common, with many possible causes (Hahn & Maron, 2016; Peters et al., 2018). Our research has uncovered several important drivers in suburban forests, but other factors that were not considered in our models also must be influential and are important for future study (e.g., plant‐soil feedbacks, Huberty et al., 2020).

## 5 CONCLUSIONS

Suburban forests are important sites for biodiversity, but many have experienced steep declines in step with deer overabundance and nonindigenous plant invasions. Here, we showed that these two common stressors can increase juvenile trees’ secondary chemicals involved in defense and stress responses. Deer generally had stronger and/or more consistent effects than stiltgrass and in some cases, their combination increased the chemical responses. The SEM analysis revealed additional, important influences on the trees’ secondary chemistry. The ecological implications for each species—and the overall suburban forest community—will depend on the relative costs and benefits to each species in their particular environmental contexts, which is a goal for future research in this area.

## CONFLICT OF INTEREST

There are no competing interests.

## AUTHOR CONTRIBUTIONS

**Janet A. Morrison:** Conceptualization (equal); Data curation (lead); Formal analysis (lead); Funding acquisition (lead); Investigation (equal); Methodology (equal); Project administration (lead); Resources (equal); Supervision (equal); Validation (equal); Visualization (lead); Writing – original draft (lead); Writing – review & editing (equal). **Bernadette Roche:** Conceptualization (equal); Data curation (supporting); Formal analysis (supporting); Investigation (equal); Methodology (equal); Project administration (supporting); Resources (equal); Supervision (equal); Validation (equal); Visualization (supporting); Writing – original draft (supporting); Writing – review & editing (equal). **Maren Veatch‐Blohm:** Data curation (supporting); Investigation (equal); Methodology (equal); Project administration (supporting); Resources (equal); Supervision (equal); Validation (equal); Writing – original draft (supporting); Writing – review & editing (supporting).

## Data Availability

Data used for this paper are available from the Dryad Digital Repository: https://doi.org/10.5061/dryad.dbrv15f38.
